# Numerical Simulation of PFRC Fracture Subjected to High Temperature by Means of a Trilinear Softening Diagram

**DOI:** 10.3390/ma16176048

**Published:** 2023-09-03

**Authors:** Fernando Suárez, Alejandro Enfedaque, Marcos G. Alberti, Jaime C. Gálvez

**Affiliations:** 1 Departamento de Ingeniería Mecánica y Minera, Universidad de Jaén, 23071 Jaén, Spain; fsuarez@ujaen.es; 2 Departamento de Ingeniería Civil-Construcción, Universidad Politécnica de Madrid, E.T.S.I. Caminos, Canales y Puertos, 28040 Madrid, Spain; alejandro.enfedaque@upm.es (A.E.); marcos.garcia@upm.es (M.G.A.)

**Keywords:** high temperature, polyolefin fibre reinforced concrete, trilinear softening function, cohesive model

## Abstract

Fibre-reinforced concrete (FRC) has been used for decades in certain applications in the construction industry, such as tunnel linings and precast elements, but has experienced important progress in recent times, boosted by the inclusion of guidelines for its use in some national and international standards. Traditional steel fibres have been studied in depth and their performance is well-known, although in recent years new materials have been proposed as possible alternatives. Polyolefin macro-fibres, for instance, have been proven to enhance the mechanical properties of concrete and the parameters that define their behaviour (fibre length, fibre proportion or casting method, for instance) have been identified. These fibres overcome certain traditional problems related to steel fibres, such as corrosion or their interaction with magnetic fields, which can limit the use of steel in some applications. The behaviour of polyolefin fibre-reinforced concrete (PFRC) has been numerically reproduced with success through an embedded cohesive crack formulation that uses a trilinear softening diagram to describe the fracture behaviour of the material. Furthermore, concrete behaves well under high temperatures or fire events, especially when it is compared with other construction materials, but the behaviour of PFRC must be analysed if the use of these fibres is to be extended. To this end, the degradation of PFRC fracture properties has been recently experimentally analysed under a temperature range between 20 °C and 200 °C. As temperature increases, polyolefin fibres modify their mechanical properties and their shape, which reduce their performance as reinforcements of concrete. In this work, those experimental results, which include results of low (3 kg/m^3^) and high (10 kg/m^3^) proportion PFRC specimens, are used as reference to study the fracture behaviour of PFRC exposed to high temperatures from a numerical point of view. The experimental load-deflection diagrams are reproduced by modifying the trilinear diagram used in the cohesive model, which helps to understand how the trilinear diagram parameters are affected by high temperature exposure. Finally, some expressions are proposed to adapt the initial trilinear diagram (obtained with specimens not exposed to high temperature) in order to numerically reproduce the fracture behaviour of PFRC affected by high temperature exposure.

## 1. Introduction

Concrete is a building material usually reinforced with steel rebars, which confer a good tensile strength to a structural element. This makes reinforced concrete one of the most versatile materials in building and civil engineering works. Nevertheless, in the last decades, fibres have become an interesting complement to steel rebars as a reinforcement and there are a wide variety of fibres that can be added, depending on the properties that need to be enhanced.

When strength needs to be improved, steel fibres have been traditionally employed and there is considerable research on their usage [1,2,3,4,5]. Nevertheless, in more recent times, polymer fibres have arisen as a competitive alternative to steel fibres, since they overcome some limitations inherent to steel fibres, such as corrosion and other durability issues [6]. Recent standards have included guidelines for using fibres in structural concrete [7,8,9,10] and provide good guidance for steel-fibre-reinforced concrete.

On another note, the influence of high temperatures on the mechanical properties of concrete is also of paramount importance in some applications. The use of reinforced concrete in nuclear reactors boosted the interest in how high temperatures and fire affect concrete mechanical performance [11]. This interest has naturally extended to fibre-reinforced concrete and has produced interesting findings depending on the fibres employed. For example, using polypropylene fibres in certain dosages reduces the risk of explosive spalling in concrete [12,13]. These fibres melt at high temperatures and generate a capillary network that relieves inner pressure inside concrete and prevents spalling. This effect has also been recently observed with recycled tyre polymer fibres [14,15]. Regarding the enhancement of concrete tensile strength, steel fibres have traditionally proven to provide good results in structural applications [16,17], in fact, if certain requirements are met, some standards permit to reduce or even eliminate reinforcing steel rebars. However, steel-fibre-reinforced structural elements exhibit explosive spalling under high temperatures, even with low fibre dosages. The use of mixed dosages, combining steel and polypropylene fibres, have successfully overcome this problem [18,19].

Regarding other types of fibres, polyolefin macro-fibres have proven to provide remarkable structural properties to concrete. Polyolefin-fibre-reinforced concrete (PFRC) has been extensively studied in recent times and its performance in vibrated or self-compacting concrete as well as the influence of many parameters on the PFRC mechanical properties or their performance under shear stresses are already well-known [20,21,22,23,24]. Nevertheless, if polyolefin fibres are to be widely employed as structural reinforcements, their performance under high temperatures must be understood; this has been recently analysed and the mechanical behaviour of PFRC specimens subjected to high temperatures has been experimentally identified [25].

From the numerical point of view, concrete fracture has long been successfully simulated using cohesive formulations, which relies on a softening diagram that drives the fracture process by progressively reducing cohesive forces between both cracking surfaces as they move apart from each other. PFRC fracture has been successfully reproduced using this approach by means of an embedded fracture model that had been used in the past with concrete and masonry [26]. To adapt the cohesive formulation to fibre-reinforced concrete, the traditional concrete softening diagram, usually expressed by a stress-crack opening relation mathematically expressed by exponential, linear or bilinear functions [27], was turned into a trilinear diagram that could reproduce the effect of fibres, extending the mechanical behaviour of the material and also correctly reproducing the load increase after cracking due to the action of fibres. The points of the trilinear diagram can be obtained with measurable values such as the volume of fibres in the mix, the fibre orientation factor or the ultimate tensile strength of the fibres [28].

The present study seeks to adapt the trilinear softening cohesive model to account for high-temperature exposure of the PFRC elements. To do this, the results obtained in [25] are used as a benchmark. These results correspond to PFRC mixes of two fibre dosages (3 and 10 kg/m${}^{3}$) exposed to increasing temperatures ranging from 20 °C to 200 °C and tested with a three-point bending flexural test. In Section 2, the experimental benchmark is briefly described, summarizing the relevant aspects of the study carried out in [25]. In Section 3, the numerical simulation is presented by shortly outlining the embedded cohesive formulation employed and the finite element model used. In Section 4, the numerical results are compared with the experimental benchmark and a modification of the trilinear diagram obtained for room temperature (20 °C) is proposed for high temperature exposure.

## 2. PFRC Performance at High Temperature

The experimental results used here as a reference correspond to an experimental campaign that can be consulted in [25]. Two sets of specimens are compared, all of them manufactured with the same concrete formulation, only modifying the fibre content, with one of the sets containing a fibre dosage of 3 kg/m${}^{3}$ (HF3) and the other one containing 10 kg/m${}^{3}$ (HF10). The formulation of each mix can be seen in Table 1.

**Figure 1:**
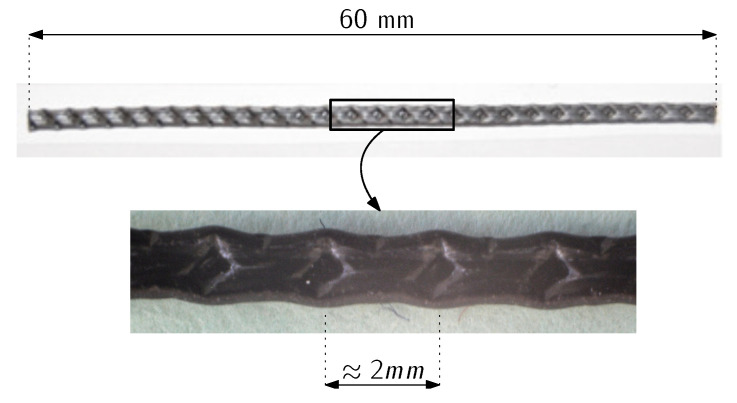
Appearance and sizes of the polyolefin fibres used.

All specimens were manufactured with 60 mm long fibres, the appearance of these fibres as well as their properties can be seen in Figure 1 and Table 2.

After manufacturing, all the specimens were introduced in a convection oven at a heating rate of 2.80 °C/min, remaining at the desired temperature for 3 h before progressively cooling for 7 h inside the oven. The bending tests were performed once the cooling process was completed.

In the case of HF3 specimens, three temperatures were used for characterisation: room temperature (20 °C), 150 °C and 200 °C, while in the case of HF10 specimens, six temperatures were chosen: room temperature (20 °C), 150 °C, 165 °C, 175 °C, 185 °C and 200 °C. Temperatures over 150 °C correspond to values at which fibres showed progressive degradation due to heating (see [25]).

**Figure 2:**
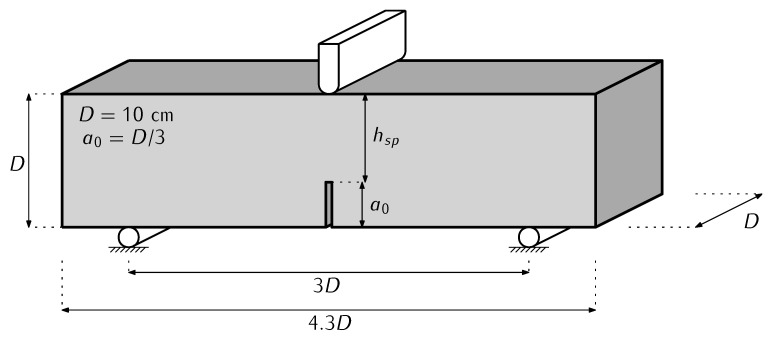
Three-point bending test specimen suggested by RILEM (adapted from [29]).

The mechanical behavior of the specimens was analysed with a three-point bending test as described by the standard RILEM TC-187-SOC [29] with notched specimens, the proportions of these specimens are shown in Figure 2.

## 3. Numerical Simulation of Fracture

This section presents the numerical work carried out to reproduce the experimental diagrams obtained for the HF3 and HF10 specimens. These simulations use an embedded crack model that is briefly described in Section 3.1, then, in the second part, the finite element models are presented. Given that the model is not new, the fracture model is very concisely described; the interested reader can find a more detailed description in [26,30].

### 3.1. Embedded Crack Model

A fracture is reproduced with the finite element method (FEM) using an element formulation that is based on the cohesive zone concept developed by Hillerborg [31]. This formulation constitutes a strong discontinuity approach that, although initially designed for concrete [26,30], has also been adapted for other types of materials, including the PFRC [28]. This model is usually described as a central forces model, given that it assumes that the cohesive stress vector t is parallel to the crack displacement vector w:

(1) $$t=\frac{f(\tilde{w})}{\tilde{w}}w\;\;\mathrm{where}\;\tilde{w}=\max\left(\left|w\right|\right)$$

with $f(\left|\tilde{w}\right|)$ representing the material softening function, that is described in terms of an equivalent crack opening, $\tilde{w}$, which identifies the maximum value of the crack opening up to that instant, which allows accounting for loading-unloading scenarios.

**Figure 3:**
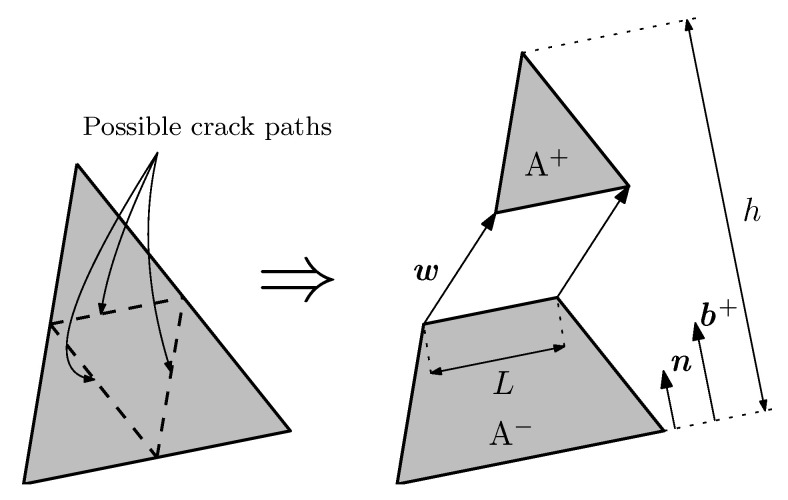
Embedded cohesive crack element.

This formulation can only be used in constant strain triangular elements, which implies using a single integration point per element, and only allows three crack orientations, which are parallel to the sides of the element (see Figure 3).

At the onset of fracture, the element is divided into parts $A^{+}$ and $A^{-}$, as shown in Figure 3. The stress vector t is obtained with (2), where *A* stands for the element surface area, *h* for the height of the triangle with respect to the opposite side of the solitary node, σ is the stress tensor, *L* is the crack length and n is the unit vector perpendicular to the crack. In order to satisfy both global and local equilibrium, the crack must be placed at mid height and must be parallel to one of the sides of the triangle, thus (2) becomes t=σ·n. A more detailed description of this formulation can be found in [30].

(2) $$t=\frac{A}{hL}\sigma \cdot n$$

The crack displacement vector w is obtained by an iterative process through Newton-Raphson’s method with given nodal displacements using expression (3). To do this, the elastic prediction of the stress tensor ($E:\epsilon^{a}$) is corrected by an inelastic stress ($E:{\left(b^{+}⊗w\right)}^{S}$). In this expression, E represents elastic tangent tensor, $\epsilon^{a}$ the strain vector that is obtained with given nodal displacements under elastic assumptions and $b^{+}$ is the gradient vector that identifies the shape function of the node at $A^{+}$ (the solitary node), which is obtained with (4). Superscript *S* denotes the symmetric part of the resulting tensor.

(3) $$\sigma =E:\left[\epsilon^{a}-{\left(b^{+}⊗w\right)}^{S}\right]$$

(4) $$b^{+}=\frac{1}{h}n$$

By using the expression of t (1) and the expression of boldsymbolσ (3), the following equality can be written:

$$\frac{f(\tilde{w})}{\tilde{w}}w=\left[E:\epsilon^{a}\right]\cdot n-\left[E:{\left(b^{+}⊗w\right)}^{S}\right]\cdot n$$

which can be also expressed as

(5) $$\left[\frac{f(\tilde{w})}{\tilde{w}}1+n\cdot E\cdot b^{+}\right]\cdot w=\left[E:\epsilon^{a}\right]\cdot n$$

where 1 represents the identity tensor.

### 3.2. Trilinear Softening Diagram

**Figure 4:**
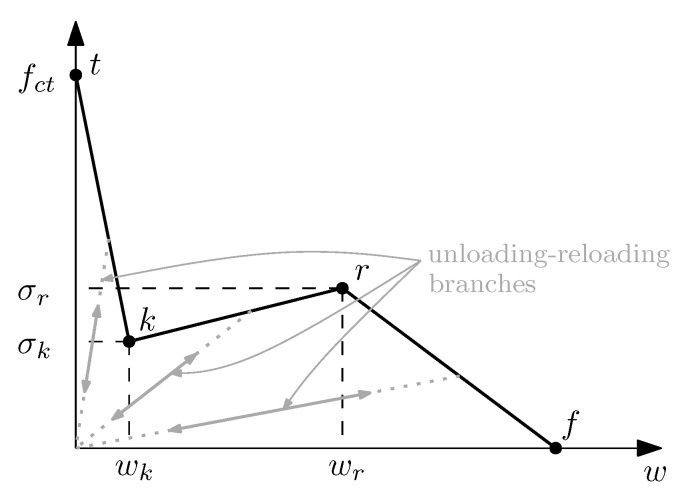
Trilinear softening diagram used to reproduce fracture in PFRC.

As proven in previous works [28,32], the above-described model can be adapted to reproduce PFRC behaviour by using a trilinear softening diagram as presented in Figure 4. Unloading and reloading branches lead to the origin and the softening function is defined by four points (*t*, *k*, *r* and *f*). This trilinear diagram can be expressed with the following equations (note that σ (bold) in (3) stands for the stress tensor while σ (not bold) represents a stress scalar value, as in (6)):

(6) $$\sigma =\left\{\begin{array}{cc} f_{ct}+\left(\frac{\sigma_{k}-f_{ct}}{w_{k}}\right)\cdot w & \mathrm{if}\;0<w\leq w_{k}\\ \sigma_{k}+\left(\frac{\sigma_{r}-\sigma_{k}}{w_{r}-w_{k}}\right)\cdot \left(w-w_{k}\right) & \mathrm{if}\;w_{k}<w\leq w_{r}\\ \sigma_{r}+\left(\frac{-\sigma_{r}}{w_{f}-w_{r}}\right)\cdot \left(w-w_{r}\right) & \mathrm{if}\;w_{r}<w\leq w_{f}\\ 0 & \mathrm{if}\;w>w_{f} \end{array}\right.$$

### 3.3. FEM Models

**Figure 5:**
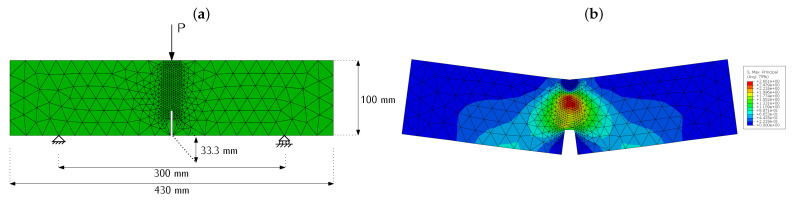
(**a**) FEM model used in the numerical simulation and (**b**) map of maximum principal stresses in one of the models at an intermediate stage of the simulation.

The three-point bending test on notched specimens shown in Figure 2 is numerically reproduced in 2D using plane stress conditions, thus a bidimensional mesh is employed. This mesh is finer between the notch tip and the applied load, since that is the region where fracture evolves; mesh refinement has been validated in previous works [32]. Figure 5a shows an image of the finite element mesh used in the models with sizes and boundary conditions. Note that, differently from the experimental setup shown in Figure 2, the numerical model includes a horizontal displacement restriction at the left support—this is needed in order to define a statically determinate structure that can be numerically solved but does not affect the performance of the model.

## 4. Definition of the Trilinear Softening Diagrams and Results

In this section, trilinear diagrams for each mix and high-temperature exposure are obtained. To do this, the coordinates of all four points *t*, *k*, *r* and *f* observed in Figure 4 must be defined. This is conducted by a trial and error process as in previous works, such as [32], and in order to keep a coherent approach as temperature increases, the specific criteria that has been employed is defined here.

Regarding point *t*, since the elastic modulus of concrete is much higher than the elastic modulus of the fibres, and given that the volume of fibres is almost negligible if compared with concrete, this point is defined as the tensile strength of concrete. Following the experimental observations, this value will be gradually reduced as temperature increases, thus reproducing a gradual material degradation. Point *k* is related to the crack opening value at which fibres start to transmit meaningful stresses across the fracture plane. The reinforcing action of fibres stops the load drop and can even lead to a load recovery. Both coordinates of point *k* have been estimated considering that the contribution of fibres is negligible up to this instant, therefore, they have been obtained with the softening diagram of a plain concrete (without fibres). The reinforcing capacity of fibres decays when a certain crack opening is reached, which is related to point *r* in the trilinear diagram. On the one hand, the abscissa $w_{r}$ is defined as a fixed value equal to 2.25 mm, based on previous experiences [32], on the other hand, the ordinate of this point, $\sigma_{r}$, is assumed to decay as temperature increases, since the experimental observations prove that high temperatures degrade the fibres material and their bonding with concrete. Point *f* identifies the crack opening value at which fibre reinforcement is negligible, this point depends on the length of the fibres and, in this case, using previous experiences, has been fixed with a value of 7.5 mm for all temperatures.

**Figure 6:**
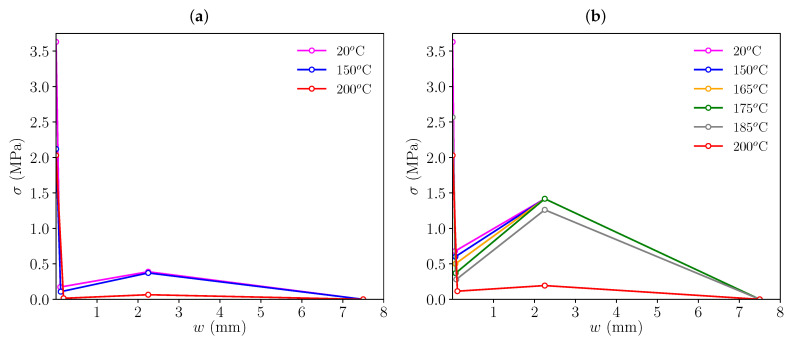
Trilinear softening diagrams used for simulating PFRC fracture specimens subjected to different high-temperature conditions: (**a**) HF3 specimens, (**b**) HF10 specimens.

**Figure 7:**
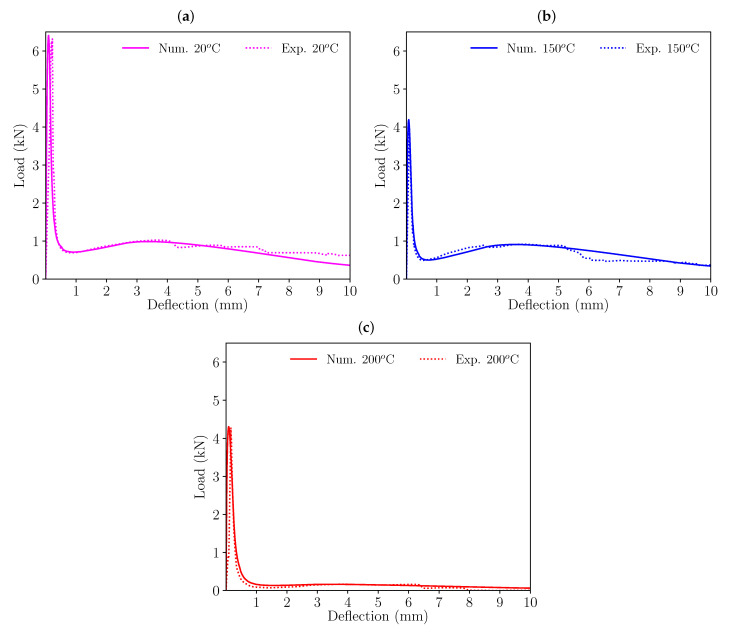
Comparison of the load-deflection diagrams obtained numerically and experimentally with HF3 specimens: (**a**) 20 °C, (**b**) 150 °C, and (**c**) 200 °C.

**Figure 8:**
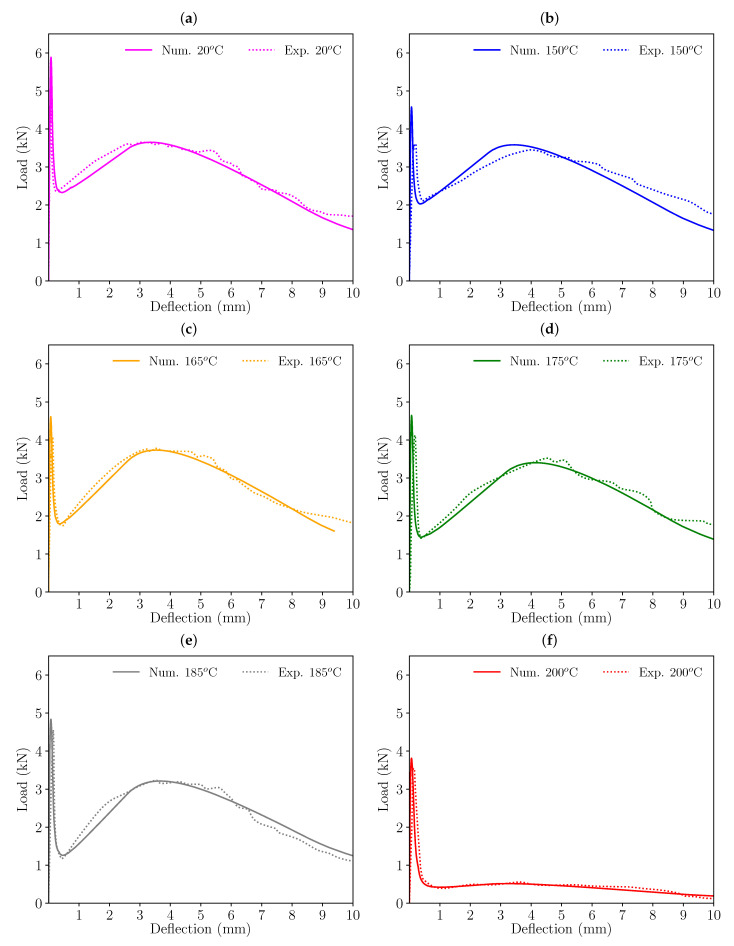
Comparison of the load-deflection diagrams obtained numerically and experimentally with HF10 specimens: (**a**) 20 °C, (**b**) 150 °C, (**c**) 165 °C, (**d**) 175 °C, (**e**) 185 °C, and (**f**) 200 °C.

Following these criteria, the initial softening diagram, adjusted for room temperature (20 °C), was modified so that the load-deflection diagrams fitted the experimental results. Figure 6 shows the resulting trilinear softening diagrams obtained for each set of specimens and temperature (the coordinates of these points for each trilinear diagram can be seen in Table 3). Figure 7 and Figure 8 presents the resulting load-deflection diagrams obtained in each case, compared with the experimental results. These diagrams are plotted with the same colour scheme used in Figure 6 for each temperature and the numerical results (solid lines) are compared with the experimental curves (dotted lines). The significant differences in the diagrams of the 200 °C case in comparison with those corresponding to lower temperatures will be discussed later.

In the case of HF3, the initial peak load is correctly reproduced for all three temperatures. The same can be noted in regards to the minimum load after peak and the subsequent remnant peak load that takes place around a deflection of 4 mm for all temperatures.

In the case of HF10, the minimum load after peak and the subsequent remnant peak load are well-reproduced for all temperatures. In some cases, especially in the case of *T* = 150 °C, the peak load in the numerical model takes place at a slightly earlier deflection than in the experimental curve. Nevertheless, in the authors’ opinion this does not make the numerical simulation inaccurate, since only one experimental value for each temperature is available and, as past experience shows, if a higher number of specimens had been tested for each case, the experimental envelopes would fit all numerical results.

**Figure 9:**
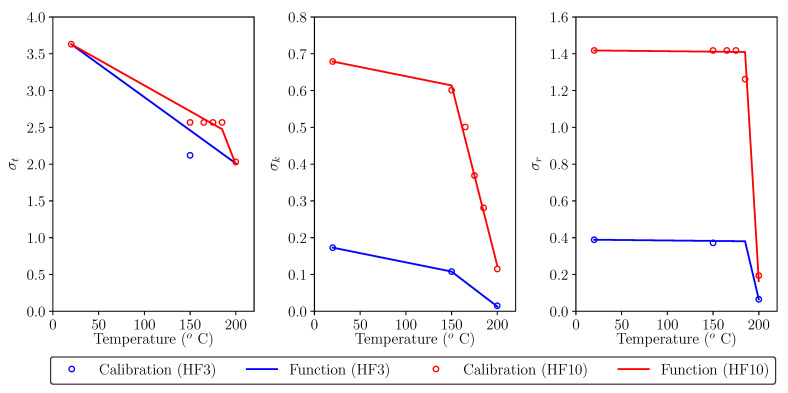
Fit of the approximation functions (7)–(9) proposed for $\sigma_{t}$ (**left**), $\sigma_{k}$ (**center**) and $\sigma_{r}$ (**right**) compared with their calibrated values.

These results show that below 150 °C, the softening diagram is not affected by high temperature exposure and, between 150 °C and 200 °C, $\sigma_{t}$, $\sigma_{k}$ and $\sigma_{r}$ must be adapted for a good numerical simulation of fracture. In all three cases, points *t*, *k* and *r* reduce their values as the temperature increases, which can be a consequence of moderate concrete and fibre degradation. In the case of $\sigma_{t}$, all specimens tested after high temperature exposure show smaller values, especially when exposed to higher temperatures, but there is not a clear trend, since in the case of HF10, values of temperatures between 150 °C and 200 °C present an unclear evolution of the peak load. For example, the peak load experimentally measured at *T* = 150 °C is smaller than at *T* = 20 °C, which would suggest a decrease as the temperature increases, but peak loads at 165 °C and 175 °C and 185 °C are higher than at 150 °C (see calibrated dots in Figure 9). With these results, temperature seems to reduce the peak load, but it is not possible to conclude how temperature affects it; a higher number of experimental results would help to provide a better conclusion. Nevertheless, the material behaviour after the initial peak is what is particularly representative of the fibre reinforcement, which is characterized by points *k* and *r*, which show a clear trend as will be discussed hereunder. Since HF3 values are limited to only two high temperature results, HF10 values will be used for the following discussion; observations are equally valid for HF3 results.

If the load drop after peak is analysed, for which $\sigma_{k}$ is the most relevant parameter, it becomes higher as temperature increases. As observed in [25], polyolefin fibres exposed to high temperature become degraded and present lower mechanical properties, especially above 100 °C. In this case, these results suggest that high temperature exposure reduce $\sigma_{k}$, especially above 150 °C.

If the remnant load is analysed, which is strongly influenced by $\sigma_{r}$, high temperature seems to be only relevant at temperatures over 185 °C, since a fixed value of $\sigma_{r}=1.418$ provides good numerical representation of fracture up to a temperature exposure of 175 °C. For temperature values over 185 °C, $\sigma_{r}$ rapidly decreases, presenting a dramatic drop at 200 °C.

### Proposed Modification of the Trilinear Diagram to Include the Effect of High Temperature Exposure

Once the fracture behaviour is correctly captured by a trilinear diagram obtained with specimens manufactured according to the usual standards, their trilinear softening diagram can be adapted for accounting of possible high temperature exposure by modifying $\sigma_{t}$, $\sigma_{k}$ and $\sigma_{r}$ values with expressions (7)–(9).

(7) $$\sigma_{t}=\sigma_{t,20}-\langle T-20\rangle \cdot A_{t}-\langle T-185\rangle \cdot B_{t}$$

(8) $$\sigma_{k}=\sigma_{k,20}-\langle T-20\rangle \cdot A_{k}-\langle T-150\rangle \cdot B_{k}$$

(9) $$\sigma_{r}=\sigma_{r,20}-\langle T-20\rangle \cdot A_{r}-\langle T-185\rangle \cdot B_{r}$$

where $\sigma_{t,20}$, $\sigma_{k,20}$ and $\sigma_{r,20}$ stand for the values of $\sigma_{t}$, $\sigma_{k}$ and $\sigma_{r}$ in the trilinear diagram calibrated at room temperature, *T* is the high temperature at which the material is exposed, $A_{t}$, $B_{t}$, $A_{k}$, $B_{k}$, $A_{r}$ and $B_{r}$ are constant values that depend on the fibre proportion of the PFRC (they are defined for 3 and 10 kg/m${}^{3}$ in Table 4 and could be interpolated for values ranging between 3 and 10) and, finally, $\langle \cdot \rangle$ denotes the Macaulay brackets:

$$\langle x\rangle =\left\{\begin{array}{cc} 0 & \mathrm{if}\;x<0\\ x & \mathrm{if}\;x\geq 0 \end{array}\right.$$

Figure 9 compares the diagrams provided by this expressions (solid lines) with the values obtained after calibrating the experimental results with the trilinear diagram (see Table 3). Values of HF3 specimens are presented in blue while those corresponding to HF10 specimens are shown in red.

While expressions (8) and (9) provide a good approximation for $\sigma_{k}$ and $\sigma_{r}$, expression (7) seems to be less accurate. As mentioned before, the available experimental results are limited and additional experimental results would help defining a reliable function for $\sigma_{t}$. In any case, the initial peak load is not so dependent on the fibre reinforcement, since the material behaviour up to that point depends almost completely on the concrete matrix, not on the fibres. In fact, the effect of fibre reinforcement is particularly relevant after the peak load and the subsequent behaviour is mainly dependent on $\sigma_{k}$ and $\sigma_{r}$, and these values can be reliably obtained with the proposed expressions. As can be observed, these proposed functions are defined as bilinear since, as mentioned before, the callibrated values of $\sigma_{k}$ and $\sigma_{r}$ dramatically decreased at a certain temperature (150 °C in the former case and 185 °C in the latter).

## 5. Conclusions

This work has studied how high temperature exposure affects the parameters of a trilinear softening diagram that allows for numerically reproducing the behaviour of PFRC specimens through a cohesive model. To do this, the experimental results of [25] have been used as reference and an embedded cohesive crack fracture model was employed, with the softening behaviour defined by a trilinear diagram. The main conclusions of this work can be summarised as follows:

- A trilinear diagram calibrated with specimens not exposed to high temperature can be adapted for high temperature exposure by modifying the ordinates of points *t*, *k* and *r* of the trilinear diagram ($\sigma_{t}$, $\sigma_{k}$ and $\sigma_{r}$ values), which decrease as temperature exposure increases.
- The load drop after the initial peak becomes higher as temperature increases. This drop is relevant beginning from a temperature exposure of 150 °C. A function for defining $\sigma_{k}$, which is the most relevant parameter to correctly model this load drop, is proposed. This function is bilinear and the decrease of $\sigma_{k}$ is higher for temperature values over 150 °C, as observed in the calibrated results of this parameter.
- The load drop of the remnant peak load that takes place after the initial peak load can be only observed for temperatures over 175 °C. A function for $\sigma_{r}$, the most relevant parameter for correctly capturing this remnant peak load, is proposed. This function is also bilinear and the decrease of $\sigma_{r}$ becomes higher at temperature values over 175 °C, in agreement with the calibrated results.

## Figures and Tables

**Table 1:** Mix proportions used in the experimental campaign [25].

	Cement (kg/m${}^{3}$)	Limestone (kg/m${}^{3}$)	Water (kg/m${}^{3}$)	Sand (kg/m${}^{3}$)	Gravel (kg/m${}^{3}$)	Grit (kg/m${}^{3}$)	Superplasticiser (% Cement Weight)	Polyolefin Fibres (kg/m${}^{3}$)
HF3	375	100	187.5	916	300	450	0.75	3
HF10	375	100	187.5	916	300	450	0.75	10

**Table 2:** Properties and dimensions of the polyolefin fibres.

Density (g/cm${}^{3}$)	Length (mm)	Eq. Diameter (mm)	Tensile Strength (MPa)	Elastic Modulus (GPa)
0.91	60	0.92	>500	>9

**Table 3:** Coordinates of *t*, *k*, *r* and *f* points of the trilinear softening diagrams shown in Figure 6.

HF3				HF10						
	**20 °C**	**150 °C**	**200 °C**		**20 °C**	**150 °C**	**165 °C**	**175 °C**	**185 °C**	**200 °C**
$w_{t}$	0.000	0.000	0.000	$w_{t}$	0.000	0.000	0.000	0.000	0.000	0.000
$\sigma_{t}$	3.630	2.120	2.030	$\sigma_{t}$	3.630	2.567	2.567	2.567	2.567	2.030
$w_{k}$	0.102	0.113	0.182	$w_{k}$	0.058	0.067	0.070	0.074	0.090	0.116
$\sigma_{k}$	0.173	0.108	0.015	$\sigma_{k}$	0.679	0.601	0.501	0.369	0.281	0.115
$w_{r}$	2.250	2.250	2.250	$w_{r}$	2.250	2.250	2.250	2.250	2.250	2.250
$\sigma_{r}$	0.389	0.372	0.065	$\sigma_{r}$	1.418	1.418	1.418	1.418	1.262	0.194
$w_{f}$	7.500	7.500	7.500	$w_{f}$	7.500	7.500	7.500	7.500	7.500	7.500
$\sigma_{f}$	0.000	0.000	0.000	$\sigma_{f}$	0.000	0.000	0.000	0.000	0.000	0.000

**Table 4:** Constant values used in expressions (7)–(9).

Fibre Content (kg/m${}^{3}$)	$A_{t}$	$B_{t}$	$A_{k}$	$B_{k}$	$A_{r}$	$B_{r}$
3	0.0007	0.025	0.0005	0.0014	0.00005	0.021
10	0.0009	0	0.0005	0.0093	0.00005	0.083

## Data Availability

No new data were created or analyzed in this study. Data sharing is not applicable to this article.
